# URBAN ENVIRONMENT EXPOSURES AND COGNITIVE HEALTH: AN EVIDENCE GAP MAP OF SYSTEMATIC REVIEWS

**DOI:** 10.1093/geroni/igae098.1249

**Published:** 2024-12-31

**Authors:** Claire Cleland, Ruth Hunter

**Affiliations:** Centre for Public Health, Queen’s University Belfast; Queen’s University Belfast, Belfast, Northern Ireland, United Kingdom

## Abstract

With a rapidly ageing global population and continued trends in urbanisation, it is crucial we understand the influence of the urban environment on cognitive health. To address gaps in the evidence, we must first understand the current evidence base. The aim of the current study was to create an Evidence Gap Map (EGM) that identifies and presents all systematic reviews investigating urban environment exposure and its impact on cognitive health outcomes. A total of 57 reviews (with 257 outcomes) were included in the EGM. Five reviews were classified as high quality, 43 as moderate quality and nine as low quality. The EGM indicates that the evidence base is concentrated on ‘environmental by-products’ and ‘social environment’ and their impact on outcomes of dementia, Alzheimer’s disease, and cognitive impairment. Areas also saturated were air pollution within ‘environmental by-products’ and educational attainment within the ‘social environment’. Areas with limited evidence included exposure to ‘traffic-related behaviours and all cognitive outcomes, and all four exposures (‘environmental by-products’, ‘social environment’, ‘urban design’ and ‘traffic related behaviours’) and child cognitive measures. The study provides an overview of the current evidence base regarding built, natural and social environmental exposures and cognitive health outcomes. The EGM provides a live, interactive, visual representation of the evidence. We identify key evidence gaps to be addressed including environmental exposures and cognitive outcomes, traffic-related behaviours, and urban by-products such as noise pollution and light pollution.

